# HCV co-infection and markers of liver injury and fibrosis among HIV-positive childbearing women in Ukraine: results from a cohort study

**DOI:** 10.1186/s12879-016-2089-7

**Published:** 2016-12-12

**Authors:** Heather Bailey, Nataliya Nizova, Violeta Martsynovska, Alla Volokha, Ruslan Malyuta, Mario Cortina-Borja, Claire Thorne, the Ukraine European Collaborative Study in EuroCoord

**Affiliations:** 1 Population, Policy and Practice Programme, UCL Great Ormond Street Institute of Child Health, University College London, 30 Guilford Street, London, WC1N 1EH UK; 2 The Public Health Center of the Ministry of Health of Ukraine, Kyiv, Ukraine; 3 Institute of Epidemiology and Infectious Diseases of NAMS, Kiev, Ukraine; 4 Shupyk National Medical Academy of Postgraduate Education, Kiev, Ukraine; 5 Perinatal Prevention of AIDS Initiative, Odessa, Ukraine

**Keywords:** HIV, Hepatitis C, Women, Liver fibrosis, Ukraine, APRI, FIB-4, Combination antiretroviral therapy, Eastern Europe

## Abstract

**Background:**

Ukraine’s injecting drug use-driven HIV epidemic is among the most severe in Europe with high burden of HCV co-infection. HIV/HCV co-infected individuals are at elevated risk of HCV-related morbidity, but little is known about burden of liver disease and associated factors in the HIV-positive population in Ukraine, particularly among women.

**Methods:**

Characteristics of 2050 HIV-positive women enrolled into the Ukrainian Study of HIV-infected Childbearing Women were described by HCV serostatus. Aspartate transaminase (AST) to platelet ratio (APRI) and FIB-4 scores were calculated and exact logistic regression models fitted to investigate factors associated with significant fibrosis (APRI >1.5) among 762 women with an APRI score available.

**Results:**

Of 2050 HIV-positive women (median age 27.7 years, IQR 24.6-31.3), 33% were HCV co-infected (79% of those with a history of injecting drug use vs 23% without) and 17% HBsAg positive. A quarter were on antiretroviral therapy at postnatal cohort enrolment. 1% of the HIV/HCV co-infected group had ever received treatment for HCV. Overall, 24% had an alanine aminotransferase level >41 U/L and 34% an elevated AST (53% and 61% among HIV/HCV co-infected). Prevalence of significant fibrosis was 4.5%; 2.5% among 445 HIV mono-infected and 12.3% among 171 HIV/HCV co-infected women. 1.2% had a FIB-4 score >3.25 indicating advanced fibrosis. HCV RNA testing in a sub-group of 56 HIV/HCV co-infected women indicated a likely spontaneous clearance rate of 18% and predominance of HCV genotype 1, with one-third having genotype 3 infection. Factors associated with significant fibrosis were HCV co-infection (AOR 2.53 95%CI 1.03-6.23), history of injecting drug use (AOR 3.51 95%CI 1.39-8.89), WHO stage 3-4 HIV disease (AOR 3.47 95%CI 1.51-7.99 vs stage 1-2 HIV disease) and not being on combination antiretroviral therapy (AOR 3.08 95%CI 1.23-7.74), adjusted additionally for HBV co-infection, smoking and age.

**Conclusions:**

Most HIV/HCV co-infected women had elevated liver enzymes and 12% had significant fibrosis according to APRI. Risk factors for liver fibrosis in this young HIV-positive population include poorly controlled HIV and high burden of HCV. Results highlight the importance of addressing modifiable risk factors and rolling out HCV treatment to improve the health outcomes of this group.

## Background

An estimated 184 million people or 2.8% worldwide are seropositive for hepatitis C virus (HCV) [1] with infections in Europe concentrated within non-EU/ EFTA countries [2]. Prevalence of anti-HCV and hepatitis B surface antigen (HBsAg) are both substantially higher among people living with HIV but vary between populations, reflecting differing modes of HIV acquisition [3]. In Eastern Europe and Central Asia, where the HIV epidemic has historically been driven by injecting drug use (IDU), around 40% of people living with HIV are HCV co-infected, accounting for over a quarter of HIV/HCV co-infections worldwide [3]. As in Western Europe, genotype (GT) 1 predominates [4].

Liver disease is a leading cause of death among HIV-positive populations, with severe immunosuppression associated with liver-related death independently of viral hepatitis co-infection [5]. HCV-related liver disease progression is accelerated in the presence of HIV co-infection [6] and the large reductions in mortality risk seen with combination antiretroviral therapy (cART) in HIV mono-infected individuals are not matched in HIV/HCV co-infected patients [7]. Although studies of HCV mono-infection have indicated slower liver disease progression in pre-menopausal women than in men, due to oestrogen-mediated and other effects [8], whether this is true for women co-infected with HIV remains unclear [9]. Other factors associated with faster liver fibrosis progression in HIV/HCV co-infection include older age at diagnosis, longer duration of infection, lower CD4 count and alcohol use [10]. HIV/HCV co-infected pregnant women have a vertical HCV transmission risk of around 10-11%, almost twice that of HCV mono-infected women [11].

Despite historically worse outcomes with interferon-based HCV treatment, HIV/HCV co-infected patients treated with directly acting antivirals (DAA) can now expect outcomes on a par with HCV mono-infected individuals [12–14]. However, the high cost of DAAs currently limits access, particularly in low and middle income countries [15].

Quantification of liver fibrosis and associated risk factors among people living with HIV is important in understanding potential future liver disease burden, to inform policies for minimising the impact of modifiable risk factors on disease progression and for identifying groups most urgently requiring HCV treatment. The FIB-4 index and the aspartate transaminase (AST) to platelet ratio (APRI) are algorithms allowing calculation of a score indicating likelihood of significant or advanced fibrosis from routinely available blood tests [16, 17]; these surrogate markers have been validated in HIV/HCV co-infected adults and are particularly useful for assessing liver fibrosis in resource-constrained settings such as Ukraine, where other non-invasive measures such as hepatic transient elastography or direct serologic markers are unavailable.

The overall aim of this study was to investigate markers of liver injury (i.e. elevated transaminases) and fibrosis in postnatal women living with HIV with and without HCV-co-infection in Ukraine. Specific objectives were to assess the prevalence of and factors associated with significant liver fibrosis, as measured by surrogate biomarkers, and to describe HCV viral characteristics (GT and viral load) for a sub-set of HIV/HCV co-infected women.

## Methods

### Study population

The Ukrainian Study of HIV-infected Childbearing Women, nested within the Ukraine European Collaborative Study (ECS), enrolled HIV-positive childbearing women with informed consent at five regional HIV/AIDS centres in Ukraine between 2007 and 2012, around 3-6 months after delivery [18]. Clinical data reported by the clinician included HIV co-infections, liver function test (LFT) and haematological test results, HIV disease status and ART. Women self-reported socio-demographic characteristics and health behaviours (including drug use and smoking). Updated clinical data were collected at routine follow-up visits. Unique study numbers were used to match records from this postnatal cohort with those in the Ukraine ECS, to obtain data on antenatal ART use and timing of HIV diagnosis [19].

As HCV GT and HCV RNA tests are not part of routine clinical care in Ukraine, information on these factors were sought as part of a sub-study from March to July 2013 in 56 HIV-positive HCV-seropositive women who had never received HCV treatment and consented for these additional tests. The 56 women were recruited at one regional centre (in Kiev), and all HCV GT and RNA tests were conducted at the same lab using real time PCR amplificator Rotor-Gene ("Corbett Research", Australia) and AmpliSense HCV-genotype-FL test kits, Russia, sensitivity 5x1000 IU/ml (1.35x10000 copies/ml).

Analyses were conducted among the 2050 women enrolled in the postnatal cohort with HCV serostatus available, and the sample of 56 women with additional HCV test results. The presence of liver fibrosis was assessed based on APRI and FIB-4.

The ECS has ethics approval from the Great Ormond Street Hospital for Children NHS Trust/Institute of Child Health Research Ethics Committee (reference 96 EB02). Ethics approval for additional HCV RNA and GT testing conducted with consent as part of this study was obtained from the UCL Research Ethics Committee (3061/002) and from the Ethics Committee of the Shupyk National Medical Academy of Postgraduate Education in Kiev.

### Definitions

APRI scores were calculated as (AST/upper limit of normal [ULN]) /platelet count x 100, with values of <0.5 indicating the absence and values of >1.5 indicating the presence of significant liver fibrosis (equivalent to METAVIR ≥ F2) [16]. FIB-4 scores were calculated using age and AST, alanine aminotransferase (ALT) and platelet levels. FIB-4 scores of <1.45 indicate absence of fibrosis and those >3.25 indicate advanced fibrosis (METAVIR ≥ F3) [17]. Scores for each measure which fell between the two cut-offs were defined as indeterminate.

Hepatitis B co-infected women were defined as those positive for HBsAg and HCV co-infected women as those with HCV antibodies. The ULN was taken as 38 U/L for AST and 41 U/L for ALT [20]; an ULN of 19 was also used to describe ALT measures, as this lower ULN may be more sensitive in identifying individuals with liver injury / hepatitis C viremia [21]. LFT elevations were categorised as mild, moderate or severe /potentially life threatening using the Division of AIDS (DAIDS) grading criteria [22]. IDU was defined by self-report, clinical assessment or neonatal abstinence syndrome in the woman’s infant (apparent in 60-80% of infants exposed to opiates in-utero [23]). Smoking status was determined at postnatal cohort enrolment.

### Selection of LFTs for analysis and calculation of APRI and FIB-4

The first eligible liver function and platelet results reported after delivery were used to calculate one APRI and one FIB-4 score for each woman; eligible test results were based on ≥1 blood samples taken within seven days. Results taken during pregnancy were excluded. Women with no LFTs reported up to their last date of follow-up or up to three years after delivery (whichever was earlier) were excluded, as were any women whose first results reported were during a subsequent pregnancy, due to the normal fluctuations in ALT, AST and platelet levels which occur during pregnancy [20] potentially affecting the validity of APRI and FIB-4. Women missing hepatitis B status (*n* = 95) were excluded from analyses of LFTs, APRI and FIB-4 scores.

### Statistical analysis

Chi-squared or Fisher’s exact tests were used for univariable comparisons of categorical variables and the Wilcoxon-Mann-Whitney rank sum test for comparing location in continuous variables. Exact logistic regression models were fitted to assess factors associated with significant fibrosis (i.e. APRI score >1.5), but not for advanced fibrosis (i.e. FIB-4 > 3.25), as the low prevalence of this more severe outcome precluded adjusted analyses. Variables associated with the outcome (*p* < 0.1) in univariable analyses were included in the multivariable model, in addition to hepatitis B co-infection status and postnatal ART use *a priori*. An interaction term was fitted to test the joint effect of IDU and HCV co-infection in a separate model. A sensitivity analysis was also conducted excluding women with a diagnosis of tuberculosis due to possible hepatic involvement. Statistical analysis was performed using STATA version 13 (Stata Corp LP, College Station USA).

## Results

A third (*n* = 677) of the 2050 HIV-positive women were HCV seropositive. Table 1 shows socio-demographic and clinical characteristics by HCV serostatus. Median age was 27.7 years (IQR 24.6-31.3). One-fifth of women had an IDU history, of whom 79% (314/398) had HCV antibodies, compared with 23% (333/1461) of those without IDU history. HCV-seropositive women were older than HCV-seronegative women (Table 1). HIV diagnosis occurred a median 16.5 months (IQR 8.6-35.9) prior to enrolment, for two-thirds during their most recent pregnancy. Almost all (96%, 1810/1879) women had received antenatal ART, with 28% (575/2029) on ART at postnatal cohort enrolment, at a median of 5.0 months (IQR 1.1-10.0) postpartum. Among the 37% (758/2050) of women with a CD4 count ≤350 cells/mm^3^ or WHO stage 3 or 4 disease (i.e. indications for treatment according to WHO guidelines at the time), postnatal treatment coverage was 61% (459/758), with no difference by HCV serostatus (χ^2^ = 0.08 *p* = 0.77). Among 512 with data available on type of ART received postnatally, 75% (*n* = 385) were on a ritonavir-boosted lopinavir-based regimen (of whom 249 received lopinavir in combination with zidovudine and lamivudine) and 17% (*n* = 86) were on a nevirapine-based regimen. Of 329 HBV infected women, 115 were on ART postnatally and 107 had information available on type of ART; among these 107, 35 (33%) were on a tenofovir-containing regimen. Of 6% (109/1783) who conceived their most recent pregnancy while on ART, almost all (*n* = 103) remained on ART postnatally. Only 1% (7/558) with data available had received HCV treatment by enrolment.

**Table 1:** Socio-demographic and clinical characteristics by HCV serostatus at postnatal cohort enrolment

	Total (*n* = 2050)	HCV antibody negative (*n* = 1383)	HCV antibody positive (*n* = 667)	*p* value
	*n* (%) or median [IQR]			
Age (*n* = 2047) (years)	27.7 [24.6, 31.3]	27.1 [24.1, 30.6]	29.1 [26.2, 32.6]	*p* < 0.01
Age at leaving full-time education (*n* = 1429)^a^				
≤ 16 years	331 (23)	159 (17)	172 (34)	*p* < 0.01
17–18 years	378 (26)	249 (27)	129 (25)	
≥ 19 years	720 (50)	515 (56)	205 (41)	
History of IDU (*n* = 1859)				
No	1461 (79)	1128 (93)	333 (51)	*p* < 0.01
Yes	398 (21)	84 (7)	314 (49)	
Ever had an IDU sex partner (*n* = 1765)				
No	1416 (80)	1025 (87)	391 (66)	*p* < 0.01
Yes	349 (20)	149 (13)	200 (34)	
History of imprisonment (*n* = 1930)				
No	1862 (96)	1274 (99)	588 (92)	*p* < 0.01
Yes	68 (4)	16 (1)	52 (8)	
Timing of HIV diagnosis (*n* = 1873)^a^				
Before most recent pregnancy	701 (37)	398 (32)	303 (48)	*p* < 0.01
During most recent pregnancy	1172 (63)	842 (68)	330 (52)	
CD4 count (*n* = 1918), (cells/mm^3^, median [IQR])	452 [324, 604]	460 [335, 609]	426 [299, 591]	*p* < 0.01
CD4 count (*n* = 1918)				
≤ 350 cells/mm^3^	566 (30)	356 (28)	210 (33)	*p* = 0.03
351-500 cells/mm^3^	582 (30)	395 (31)	187 (30)	
> 500 cells/mm^3^	770 (40)	538 (42)	232 (37)	
WHO stage (*n* = 2041)				
Stage 1	1424 (70)	1065 (77)	359 (54)	*p* < 0.01
Stage 2	269 (13)	145 (11)	124 (19)	
Stage 3	267 (13)	124 (9)	143 (21)	
Stage 4	81 (4)	41 (3)	40 (6)	
On ART (*n* = 2029)				
No	1454 (72)	1012 (74)	442 (67)	*p* < 0.01
Yes	575 (28)	357 (26)	218 (33)	
Hepatitis B co-infection (*n* = 1955)				
No	1626 (83)	1170 (86)	456 (76)	*p* < 0.01
Yes	329 (17)	184 (14)	145 (24)	
Smoking history (*n* = 2021)				
Never smoked	578 (29)	460 (34)	118 (18)	*p* < 0.01
Past smoker	484 (24)	364 (27)	120 (18)	
Current smoker, <15 cigarettes /day	417 (21)	304 (22)	113 (17)	
Current heavy smoker, ≥15 cigarettes /day	440 (22)	176 (13)	264 (40)	
Current smoker, cigarettes/day not reported	102 (5)	57 (4)	45 (7)	

^a^Available for women with matched pregnancy data from the ECS only

### HCV genotype and viral load

Among the sub-group of 56 HCV-seropositive women tested for HCV RNA and GT (median 32.0 years), IDU history was reported in 39% (21/54); none were positive for HBsAg. Ten (18%) were non-viremic for HCV. Among 46 women with detectable HCV RNA (i.e. >200 copies/ml) median viral load was log_10_ 5.58 copies/ml (IQR 5.18-6.08): 9 (20%) had GT1a, 20 (43%) GT1b, 1 uncategorised GT1 and 16 (35%) GT3a. HCV GT1 predominated among those with and without an IDU history (found in 11/18 and 18/27 respectively).

### Markers of liver injury or fibrosis

**Fig. 1:**
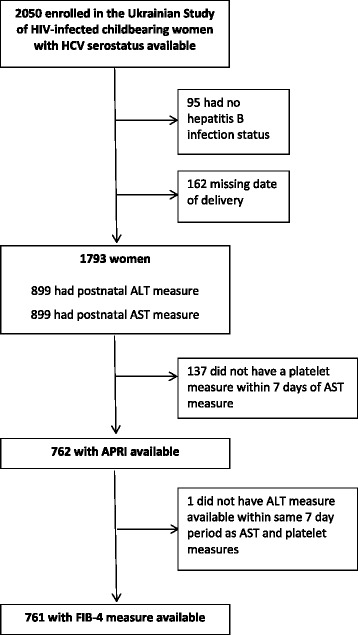
Availability of surrogate biomarkers of liver fibrosis. Footnote: The APRI /FIB-4 score was available a median of 5.9 months [IQR 3.8-7.3] after delivery, with 56% (*n* = 424) having the required blood tests score reported at postnatal cohort enrolment and 44% (*n* = 338) at a follow-up visit

Figure 1 indicates the number of women with APRI and/or FIB-4 available; this group were similar to those without APRI and/or FIB-4 in terms of HCV co-infection (30% (231/762) vs 33% (342/1031) respectively, *p* = 0.20), IDU history (20% in each group (150/753 and 203/997) *p* = 0.82) and age (Wilcoxon-Mann-Whitney rank sum test *p* = 0.66); however, women with an APRI score were more likely to have WHO stage 3-4 HIV disease than those without (20% (149/758) vs 16% (164/1030), *p* = 0.04) and HBV co-infection (19% (146/762) vs 16% (161/1031) respectively, *p* = 0.049).

Table 2 shows markers of liver injury and fibrosis by co-infection status. Among 899 women with liver enzyme data, median AST and ALT levels were 30 U/L (IQR 19-47) and 24 U/L (IQR 18-39) respectively. Among the HIV/HCV co-infected women, just over half had ALT levels above 41 U/L and 84% had a level above 19 U/L; 61% had AST above ULN of 38 U/L (Table 2). Overall 4.5% (34/762) women had an APRI score >1.5, indicating significant fibrosis, and 1% (9/761) of women had a FIB-4 score >3.25, indicating advanced fibrosis. HCV co-infected women had higher FIB-4 scores overall than those without HCV infection (Table 2, Wilcoxon-Mann-Whitney rank-sum test *p* < 0.01) and were less likely to have a FIB-4 score <1.45 (Table 2).

**Table 2:** Markers of liver injury or fibrosis by viral hepatitis co-infection status

	HIV Mono-infection	HIV/HCV Co-infection	HIV/HBV Co-infection	HIV/HCV/HBV Co-infection	Total
	*n* (%) or median [IQR]				
ALT					
Number with measurement available	514	231	91	63	899
Measure above 19 U/L	269 (52.3)	195 (84.4)	60 (65.9)	52 (82.5)	576 (64.1)
Measure above 41 U/L	64 (12.5)	122 (52.8)	18 (19.8)	11 (17.5)	215 (23.9)
DAIDS grading (ULN taken as 41 U/L)					
Grade 1, mild	33 (6.4)	72 (31.2)	9 (9.9)	10 (15.9)	124 (13.8)
Grade 2, moderate	3 (0.6)	21 (9.1)	0	0	24 (2.7)
Grade 3 or 4, severe /potentially life threatening	0	7 (3.0)	0	0	7 (0.8)
AST					
Number with measurement available	514	231	91	63	899
Measure above ULN of 38 U/L	127 (24.7)	141 (61.0)	18 (19.8)	18 (28.6)	304 (33.8)
DAIDS grading (ULN 38 U/L)					
Grade 1, mild	81 (15.8)	86 (37.2)	12 (13.2)	7 (11.1)	186 (20.7)
Grade 2, moderate	2 (0.4)	25 (10.8)	0	4 (6.4)	31 (3.5)
Grade 3 or 4, severe /potentially life threatening	1 (0.2)	4 (1.7)	0	0	5 (0.6)
APRI score					
Number with measure available	445	171	86	60	762
Median APRI [IQR]	0.27 [0.17, 0.42]	0.55 [0.30, 0.92]	0.23 [0.17, 0.34]	0.30 [0.21, 0.43]	0.30 [0.18, 0.52]
< 0.5 (no significant fibrosis)	358 (80.5)	81 (47.4)	74 (86.1)	48 (80.0)	561 (73.6)
0.5- ≤ 1.5 (intermediate)	76 (17.1)	69 (40.4)	12 (14.0)	10 (16.7)	167 (21.9)
> 1.5 (significant fibrosis)	11 (2.5)	21 (12.3)	0	2 (3.3)	34 (4.5)
FIB-4					
Number with measure available	444	171	86	60	761
Median FIB-4 [IQR]	0.61 [0.39, 0.93]	0.88 [0.59, 1.47]	0.53 [0.36, 0.74]	0.68 [0.50, 0.90]	0.67 [0.42, 1.01]
< 1.45 (no advanced fibrosis)	403 (90.8)	128 (74.9)	85 (98.8)	53 (88.3)	669 (87.9)
1.45-3.25 (intermediate)	37 (8.3)	41 (24.0)	1 (1.2)	4 (6.7)	83 (10.9)
> 3.25 (advanced fibrosis)	4 (0.9)	2 (1.2)	0	3 (5.0)	9 (1.2)

### Factors associated with significant fibrosis (APRI >1.5)

In univariable analyses, APRI score >1.5 was associated with HCV seropositivity, IDU history, more advanced HIV disease, smoking and increasing age (Table 3). In multivariable analyses adjusting for these factors plus HBV co-infection and postnatal ART, associations between significant fibrosis and HCV co-infection, IDU history and more advanced HIV disease were all attenuated (reflecting their inter-correlation) but remained statistically significant (Table 3). Smoking and age were no longer associated with risk of significant fibrosis, however women on ART were less likely to have an APRI score >1.5 than untreated women.

**Table 3:** Factors associated with APRI score >1.5

	Proportion with APRI >1.5 (significant fibrosis)	Odds ratio (*n* = 726)	*p* value	Adjusted odds ratio (*n* = 726)	*p* value
Hepatitis C antibody status					
Negative	2.1% (11/531)	1.00		1.00	
Positive	10.0% (23/231)	5.16 (2.50-10.65)	<0.001	2.53 (1.03-6.23)	0.044
Hepatitis B surface antigen					
Positive	1.4% (2/146)	1.00		1.00	
Negative	5.2% (32/616)	3.28 (0.89-12.02)	0.074	3.28 (0.85-12.71)	0.085
History of injecting drug use					
No	1.8% (11/603)	1.00		1.00	
Yes	15.3% (23/150)	9.15 (4.40-19.01)	<0.001	3.51 (1.39-8.89)	0.008
WHO stage^a^					
1–2	2.6% (16/605)	1.00		1.00	
3–4	12.4% (18/145)	5.02 (2.52-10.02)	<0.001	3.47 (1.51-7.99)	0.003
CD4 count^a^					
> 350 cells/mm^3^	3.9% (22/563)	1.00			
201-350 cells/mm^3^	5.3% (7/132)	1.46 (0.62-3.42)	0.381		
≤ 200 cells/mm^3^	8.2% (4/49)	2.32 (0.80-6.67)	0.119		
Smoker at postnatal cohort enrolment					
No	2.7% (11/409)	1.00		1.00	
Yes, current smoker	6.8% (23/340)	2.59 (1.26-5.33)	0.010	1.06 (0.46-2.42)	0.896
Age					
Per increasing year		1.10 (1.03-1.18)	0.007	1.08 (0.99-1.18)	0.070
Postnatal ART^b^					
Yes	4.0% (10/253)	1.00		1.00	
No	4.8% (24/500)	1.15 (0.55-2.41)	0.715	3.08 (1.23-7.74)	0.017

^a^Closest to timing of liver function test measures ^b^At postnatal cohort enrolment

Although IDU history and WHO stage remained independently associated with APRI score >1.5 after adjusting for HCV co-infection, there was a significant interaction between IDU history and HCV co-infection status (*p* = 0.031). Fitting the final model above, but restricted to HIV/HCV co-infected women (*n* = 219), showed that IDU was not associated with significant fibrosis (AOR 2.30, 95% CI 0.76-6.95) in this sub-group.

Eighteen women included in the main multivariable model had a tuberculosis diagnosis. In a sensitivity analysis excluding these women, the association between WHO stage 3-4 disease and APRI >1.5 remained largely unchanged (AOR 3.70 95% CI 1.56-8.78, *p* = 0.003) as did the associations between HCV co-infection and APRI score >1.5 (AOR 2.96, 95% CI 1.17-7.47, *p* = 0.022) and IDU and APRI score >1.5 (AOR 3.39, 95% CI 1.32-8.72, *p* = 0.011).

## Discussion

In this cohort of young childbearing HIV-positive women, over half of whom had been diagnosed with HIV in the preceding 18 months, a third were co-infected with HCV. Half of the HIV/HCV co-infected group had an ALT measure above 41 U/L compared with one in six of those with HIV mono-infection; the proportions with significant fibrosis (APRI score >1.5) were 12% and 2.5% respectively. Overall, 84% of HIV/HCV co-infected women had an ALT measure above 19 U/L (a level found to have 76% sensitivity in identifying HCV viraemia among 209 HCV antibody positive blood donors in Italy [21]). A more detailed characterisation of HCV RNA in a sub-group of women indicated a likely spontaneous clearance rate of around 18% (compared with 23% in a pan-European study of HIV-positive patients [24]) and a predominance of GT1, although with one-third having GT3 infection.

Factors associated with increased risk of significant fibrosis in adjusted analyses were HCV co-infection, IDU history, more advanced HIV disease, and no postnatal ART. Overall, 17% of women were HBV co-infected, increasing to 24% among those HCV-seropositive. Hepatitis B has been associated with more rapid liver fibrosis progression among HIV mono-infected and HIV/HCV co-infected individuals [25, 26] but its role in liver fibrosis progression may be complicated by potential interactions of hepatitis B and/or delta virus with HCV to suppress HCV viremia, or vice versa [27, 28]. HBsAg-positive women were less likely to have significant fibrosis than HBsAg-negative women in adjusted analyses, although this did not reach statistical significance (*p* = 0.085). Increasing age was not associated with significant fibrosis in this young cohort, with median age 28 years.

Prevalence of HCV co-infection among HIV-positive women with an IDU history here was 79%, in line with previous estimates for Ukraine [29] and more generally for IDUs in transitional countries [30]. Women with a history of IDU had increased risk of significant fibrosis even after adjusting for HCV and HBV co-infection, possibly reflecting hepatotoxic effects of injected substances. Homemade poppy straw is the drug most commonly injected in Ukraine and in this cohort [18], usually taken with a range of other drugs [31] which may cause liver transaminase elevation. Injecting drug use has itself also been associated with thrombocytopenia [32], which would result in a higher APRI score; however, APRI has been validated in HCV infected individuals among whom IDU is common.

ART coverage was suboptimal, reflecting the national situation – only 26% of adults living with HIV in Ukraine were estimated to be receiving ART in 2013 [33]; here, over a third of women with treatment indications were not on cART postnatally. Among HIV-positive people living in Eastern Europe have a higher incidence of AIDS-related death and lower incidence of liver-related death than those in the West, reflecting poorer cART coverage [34]; the proportion of deaths attributable to liver disease in Eastern Europe can be expected to rise as coverage of cART increases.

We found that women with advanced /severe HIV disease had a more than three-fold increased risk of significant fibrosis, indicating accelerated liver fibrosis associated with high HIV RNA load and/or the presence of AIDS-defining diseases with hepatic involvement [35]. Almost three-quarters had stopped antenatal ART by postnatal cohort enrolment, reflecting the national prevention of mother-to-child transmission (PMTCT) policy at the time, and untreated women were at independently increased risk of significant fibrosis. ART interruptions and/or rebounds in HIV RNA have been associated with more rapid liver fibrosis progression in HIV/HCV co-infection patients [36], and short course ART for PMTCT may be particularly detrimental for this group’s longer term prognosis. Option B+ (lifelong ART initiated in all pregnant women with HIV) became national policy in Ukraine in 2015 and may help to improve postnatal cART coverage, with concomitant reductions in liver fibrosis progression [37–39].

Among non-IDUs, the 23% HCV co-infection prevalence points to potential under-ascertainment of IDU, but also other modes of HCV acquisition including iatrogenic acquisition or household/ sexual acquisition from IDU partners. Heterosexual HCV acquisition risk may be substantially increased with HIV co-infection [40] and our study population are at high risk of other sexually transmitted infections [41] which may act to synergistically to increase risk of sexual HCV acquisition. Improved understanding of modes of HCV transmission in this population is crucial for informing prevention strategies, including reducing HCV re-infections following HCV treatment.

Other published studies of hepatic fibrosis in HIV/HCV co-infection predominantly include older individuals, the majority males and IDUs, limiting comparisons with our study. Half of 73 HIV/HCV co-infected women in the Women’s Interagency HIV Study had significant fibrosis (defined as liver stiffness ≥7.1 kPa by transient elastography) [42] while 24% of 800 HIV/HCV co-infected patients (30% female) enrolled in the Canadian Coinfection Cohort Study had an APRI score ≥1.5 [43], similar to 20% in a cohort of 116 HIV/HCV co-infected patients (36% female) in France, with median age 44 years [44]. Although the HIV/HCV co-infected women here were 13-15 years younger than individuals in these studies, 12% already had significant fibrosis. Future rates of progression will depend on modifiable risk factors, for example increasing cART and availability of HCV treatment. In the Canadian Coinfection Cohort Study, the incidence rate for progression to an APRI score ≥1.5 was 14.0/100 person-years among HIV/HCV co-infected women vs. 8.9/100 person-years among men [9], thus, the HIV/HCV co-infected women in our cohort may represent a high risk group for developing significant liver fibrosis based on their gender as well as current fibrosis rates.

HCV treatment was not publicly funded in Ukraine at the time of our study. In 2014 a national HCV treatment programme was launched based on interferon, and in 2015 this was extended to include sofosbuvir, with a Global Fund-supported pilot scheme also aiming to provide sofosbuvir treatment to 1500 patients from priority groups including those with HIV co-infection, from mid-2015 [45]. The expansion of HCV treatment access alongside high quality HIV treatment and care is crucial to prevent avoidable morbidity in HIV/HCV co-infected people in Ukraine, while in childbearing women the successful treatment of HCV could also prevent perinatal transmissions in future pregnancies.

This study had several limitations. APRI may perform less well in HIV/HCV co-infection than in HCV mono-infection, but the >1.5 cut-off used here is highly specific for significant fibrosis (0.95, 95% CI 0.92-0.97) with lower sensitivity (0.27 95% CI 0.23-0.31) [46]; the prevalence of significant fibrosis we report should therefore be considered a minimum estimate. Information on duration of diagnosed HCV was not available (age was used as a proxy) and we also lacked information on other factors relevant to liver injury, for example alcohol consumption, other medications and indicators of metabolic syndrome; body mass index was not interpretable in this cohort of recently delivered women. Elevated transaminases were based on one measure only as we lacked repeated measures, and we did not have information on delta virus which may contribute to fibrosis progression among those with hepatitis B. As HCV RNA quantification is not routine in Ukraine, our definition of HCV co-infection was based on HCV serostatus (in common with other studies of HIV/HCV co-infection [7]) and so will have included some women who had spontaneously cleared the virus; the proportion of HCV viremic women with APRI score >1.5 may therefore be higher than our estimates for the HIV/HCV co-infected group suggest. Resources to support HCV RNA and GT testing were available for 56 HIV/HCV co-infected women only as part of this study. Liver biopsy and/or transient elastography were not available to validate the non-invasive markers used in this study, reflecting lack of use / availability in participating HIV centres. Women with WHO stage 3-4 disease were more likely to have an APRI score available than those with less severe HIV disease. Women in this study were engaged with HIV care and may have a different liver fibrosis risk profile to the wider HIV-positive population.

## Conclusions

There is a high burden of HCV co-infection among childbearing women living with HIV in Ukraine, particularly among those with an IDU history. Most HIV/HCV co-infected women had elevated liver enzymes indicating liver injury, and 12% had significant fibrosis according to their APRI scores. HCV treatment rates were extremely low and a high proportion of this population of postnatal women were not on ART, a situation which is likely to have changed now that Ukraine has adopted an Option B+ policy. Our results highlight the importance of addressing modifiable risk factors and roll-out of HCV treatment for improving longer term prognosis.

## Abbreviations

(c)ART: (combination) antiretroviral therapy
ALT: Alanine aminotransferase
APRI: AST to platelet ratio
AST: Aspartate transaminase
DAIDS: Division of AIDS
ECS: European Collaborative Study
GT: Genotype
HBsAg: Hepatitis B surface antigen
HCV: Hepatitis C virus
IDU: Injecting drug use
IQR: Interquartile range
PMTCT: Prevention of mother-to-child transmission
ULN: Upper limit of normal
WHO: World Health Organisation
